# The impostor phenomenon among doctoral students: a scoping review

**DOI:** 10.3389/fpsyg.2023.1233434

**Published:** 2023-10-02

**Authors:** Yanyan Wang, Wanhe Li

**Affiliations:** School of Education, Tianjin University, Tianjin, China

**Keywords:** the impostor phenomenon, doctoral students, academic development, psychological well-being, scoping review

## Abstract

Emerging evidence suggests that the Impostor Phenomenon (IP) among doctoral students is a serious problem worldwide academic. Although previous studies demonstrate that IP can endanger doctoral students’ academic advancement and psychological well-being, limited studies systematically and comprehensively explore the IP among those population. Thus, the fundamental goal of this study is to conduct a scoping review of IP among doctoral students so as to clarify the reality of their situation. Systematic searches were conducted using 5 databases: Springer, Google Scholar, Web of Science, PubMed, and JSTOR for empirical studies published from 1978 to 2023. Two reviewers independently carried out the literature search, study selection, data extraction and assessment of study; disagreements were resolved by a third reviewer. Thirty empirical studies covering four specific domains were include in current research, including the characteristics of IP among doctoral students, factors contributing to IP among doctoral students, correlation of IP with doctoral students’ mental illness, and measurement of IP. The findings of this study may provide insight to improving the comprehension of IP among doctoral students and establishing the groundwork for future research in this field.

## 1. Introduction

Recent studies have revealed that graduate students worldwide are increasingly affected by psychological problems and their associated manifestations are a source of concern. For example, a study by the American Psychiatric Association (2017) found that 92% of graduate students felt anxious during their first year. Graduate students are at least six times more likely than the general population to suffer from depression and anxiety, with 41% of graduate students suffering from moderate to severe anxiety and 6% of the general population having such experience (Evans et al., 2018). These populations are also more likely to commit suicide. Based on a poll of 3,352 UK Ph.D. students, 33 and 35% of them met the criteria for “risk of suicide” (Hazell, 2022). This result indicates that postgraduate students’ mental health status is not promising and should be addressed carefully. Simultaneously, doctorate students are plagued by the prevalence of mental health crises, with dropout rates ranging from 30 to 50% (Stubb et al., 2012). Research from French Ph.D. students found comparable results, with 40% reporting that excessive stress was a significant factor in their decision to drop out (Van de Velde et al., 2019). In addition to focusing on aforementioned problems, recently, serious concerns have been raised about the Impostor Phenomenon (IP) in the academic field, such as increasing doubts in Ph.D. students regarding their self-identity roles and doctoral-level studies (Bothello et al., 2019).

Clance and Imes put forward the definition of the IP in 1978. They used it to designate an internal experience of intellectual phoniness which appears to be particularly prevalent and intense among a select sample of high achieving women. Despite outstanding academic and professional accomplishments, women who experience the impostor phenomenon persist in believing that they are really not bright and have fooled anyone who thinks otherwise (Clance and Imes, 1978), and maintain low expectations for their own performance, as well as for other women. They have undoubtedly been instilled with family-entrenched notions and self-consolidated preconceptions of societal gender roles (Kolligian and Sternberg, 1991). A preliminary study by Clance and Imes suggested that the occurrence of Impostor Phenomenon (IP) is less frequent in men and more widespread among high-achieving women (Clance and Imes, 1978). Indeed, subsequent studies demonstrated that IP occurs in both men and women. And compared with women, men occasionally had higher levels of IP (Topping and Kimmel, 1985). Overall, the academic community is divided on whether there are substantial gender disparities concerning IP.

The subsequent development of this phenomenon has garnered widespread attention from scholars, who believe that it encompasses a broader range of groups and involves more complex characteristics. Our study draws on the research of Nori and Vanttaja (2022), and it suggests that IP refers to a mindset in which a person considers themselves less proficient than they actually are. While the people around them might find the person skilled and competent, they themselves think that they only managed to give an impression of their prowess. Roughly speaking, impostor phenomenon is a condition suffered by people who have external markers of success, such as high grades and professional accolades, who nevertheless believe themselves to be inadequate (Hawley, 2019). Overall, IP includes three typical characteristics: fear of success, fear of failure, and low self-esteem (Neureiter and Traut-Mattausch, 2016). First, individuals with IP believe that their achievements are attributable to chance rather than hard work or their competence or intelligence. Previous achievements are nothing more than a corroboration of their intellectual deception (Harley, 2019). Consequently, they fear success since it implies sustaining the same high level of achievement in the future. Second, they are terrified of failure, which for them entails total self-denial. This causes individuals to put themselves under high pressure and anxiety, worrying that any deviation may increase the risk of failure (Chandra et al., 2019). In fact, individuals with IP fall short of expectations for their own (Clance and Imes, 1978), and are unable to mitigate all possibly uncontrolled hazards with their personal strength. Third, they may display low self-esteem and tend to focus on their negative traits, leading them to isolate themselves from others due to the belief that they are not flawless (Patzak et al., 2017).

The impostor phenomenon is common among doctoral students, as evidenced by various research. For example, 50.6% of Ph.D. students in ECOOM Ghent University believed they suffer from IP (Van de Velde et al., 2019). This statistic closely matches the findings of an experiment of 130 Romanian psychology graduate students. In Maftei et al. (2021) research, they used the Clance Impostor Phenomenon Scale (Clance, 1985) to measure IP. The 20 items contained in the scale measure each answer on a 5-point Likert scale, with 1 = not at all true, and 5 = very true. Therefore, the higher the score, the higher the chance of experiencing associated symptoms of the impostor phenomenon. When adding the scores for each answer, a score lower than or equal to 40 means that the individual has several characteristics of IP; a score between 41 and 60 suggests moderate experiences of IP, while a score between 61 and 80 suggests that the individual frequently feels the associated symptoms of the impostor phenomenon; finally, a score higher than 80 suggests intense experiences of IP. Their results indicated that 56.15% of the participants experienced high and intensity IP. More specifically, they presented high levels of psychological distress and procrastination. Similarly, in an analysis, 73% of 1,450 medical students from three Canadian institutions had moderate or severe IP symptoms (Neufeld et al., 2023). Through quantitative investigations, all of the aforementioned studies confirmed the prevalence of IP among the doctoral student population. Moreover, several in-depth qualitative studies have been conducted on this issue. For example, several studies showed that the majority of postgraduate students have imposter feelings (Cisco, 2020). Craddock et al. (2011) used a planned and standardized sample including six American doctoral students of various ages and backgrounds. Semi-structured interviews were centered on the respondents’ academic achievement, fears, and struggles. When the researchers presented the concept “IP” to the participants, they quickly identified it and exhibited an interest in sharing their experiences with IP. Overall, both quantitative and qualitative research have the comparable findings that IP exists among Ph.D. students and is not an intended consequence but has spread throughout the doctoral student population.

While highly educated individuals are often recognized for their academic achievements, society often overlooks the emotional and physical pain that they may experience. This imposter feeling had a serious impact on doctoral students’ daily life and academic performance and threatened their psychological health. Recently, there has been a growing research focus on IP. For example, some scholars have studied IP in groups, such as corporate workers, university staff members, and adolescents. Review articles have also been published on the prevalence, prediction, and treatment of IP (Bravata et al., 2019), measurement scales for IP (Mak et al., 2019), and IP in the resident population (Gottlieb et al., 2019). The objective of our study is to map the impostor phenomenon among doctoral students, aiming to understand the research progress in this field. Specifically, we aim to uncover the characteristics and quantitative trends of the impostor phenomenon among doctoral students. Additionally, we seek to explore the underlying causes of this phenomenon and gain a visual understanding of its connection with psychological issues faced by doctoral students. Moreover, we plan to compile and summarize existing measurement tools for assessing impostor phenomenon among doctoral students, with the intention of facilitating targeted evaluations in the future. Finally, we will summarize the gaps in existing studies to provide valuable insights for future research endeavors.

## 2. Methods

Scoping reviews, a type of knowledge synthesis, are now seen as a valid approach (Daudt et al., 2013). A key strength of the scoping study is that it can provide a rigorous and transparent method for mapping areas of research. In a relatively short space of time (compared with full systematic review), reviewers are in a position to illustrate the field of interest in terms of the volume, nature and characteristics of the primary research. This analysis in turn makes it possible to identify the gaps in the evidence base, as well as summarizing and disseminating research findings (Arksey and O’Malley, 2005), which is apt for our study. Given that Arksey and O’Malley have built a more classical five-step research methodology, we outlined a framework in this section based on their approach. The steps were as follows: (1) identifying the research question, (2) identifying relevant studies, (3) selecting the studies, (4) extracting and charting the data, and (5) summarizing the results (Arksey and O’Malley, 2005). We reported the study according to PRISMA-ScR (Preferred Reporting Items for Systematic Reviews and Meta-Analyses extension for Scoping Review), which was proposed by Tricco and his colleagues. The PRISMA-ScR is a checklist that consists of 7 sections and 27 items. It provides guidance for every aspect of scoping research, such as defining research questions, formulating search strategies for literature retrieval, the process of literature screening, methods of data extraction, and assessing the quality of included studies (Tricco et al., 2018). When conducting our research, we followed the guidance of PRISMA-ScR to plan, conduct, and report our scoping study. By adhering to the guidance of PRISMA-ScR, the quality and transparency of the research can be ensured.

### 2.1. Identifying the research question

In order to conduct a thorough examination of this topic, we took into account the broader context of the impostor phenomenon, including its underlying theories, conceptions, and measurement approaches. This foundational understanding provided a basis for investigating the presence of IP specifically among doctoral students. Furthermore, to ensure a comprehensive understanding, we also discussed the associations between academic performance and mental health, with a particular focus on how IP may mediate or moderate these relationships. As such, in the first step of this study, our research questions were placed into a broader domain. In general, the following five aspects were addressed: (1) What are the latest research developments in the impostor phenomenon? (2) What are the characteristics of impostor phenomenon among doctoral students? (3) Why do doctoral students experience the Impostor Phenomenon? (4) Are there any potential connections between mental health status and impostor phenomenon among doctoral students? (5) What scales are currently available to measure the impostor phenomenon?

### 2.2. Identifying relevant studies

Since scoping reviews require access to as comprehensive a literature as possible (Arksey and O’Malley, 2005), we identified several widely used electronic databases in academic research to ensure a comprehensive and up-to-date review of the existing literature, including: Springer, Google Scholar, Web of Science, PubMed, and JSTOR. We selected a combination of relevant keywords such as: “impostor phenomenon,” “psychological health,” with “doctoral students” to capture all relevant studies related to IP among doctoral students. We restricted our searches to English as it is the primary language of scholarly publication and dissemination. In addition to our initial search, we searched the reference lists of existing reviews for additional publications that may have been overlooked. We employed the following steps to search the reference lists: (1) extract the reference lists from each obtained article; (2) review the extracted reference lists and identify potentially relevant studies based on their titles and authors; (3) search in electronic databases and determine the availability and accessibility of the full texts of the identified studies; (4) assess the relevance and suitability of the studies found in the reference lists; and (5) repeat the above steps iteratively, going back and forth between the extracted reference lists and the database searches.

### 2.3. Study selection

**Figure 1:**
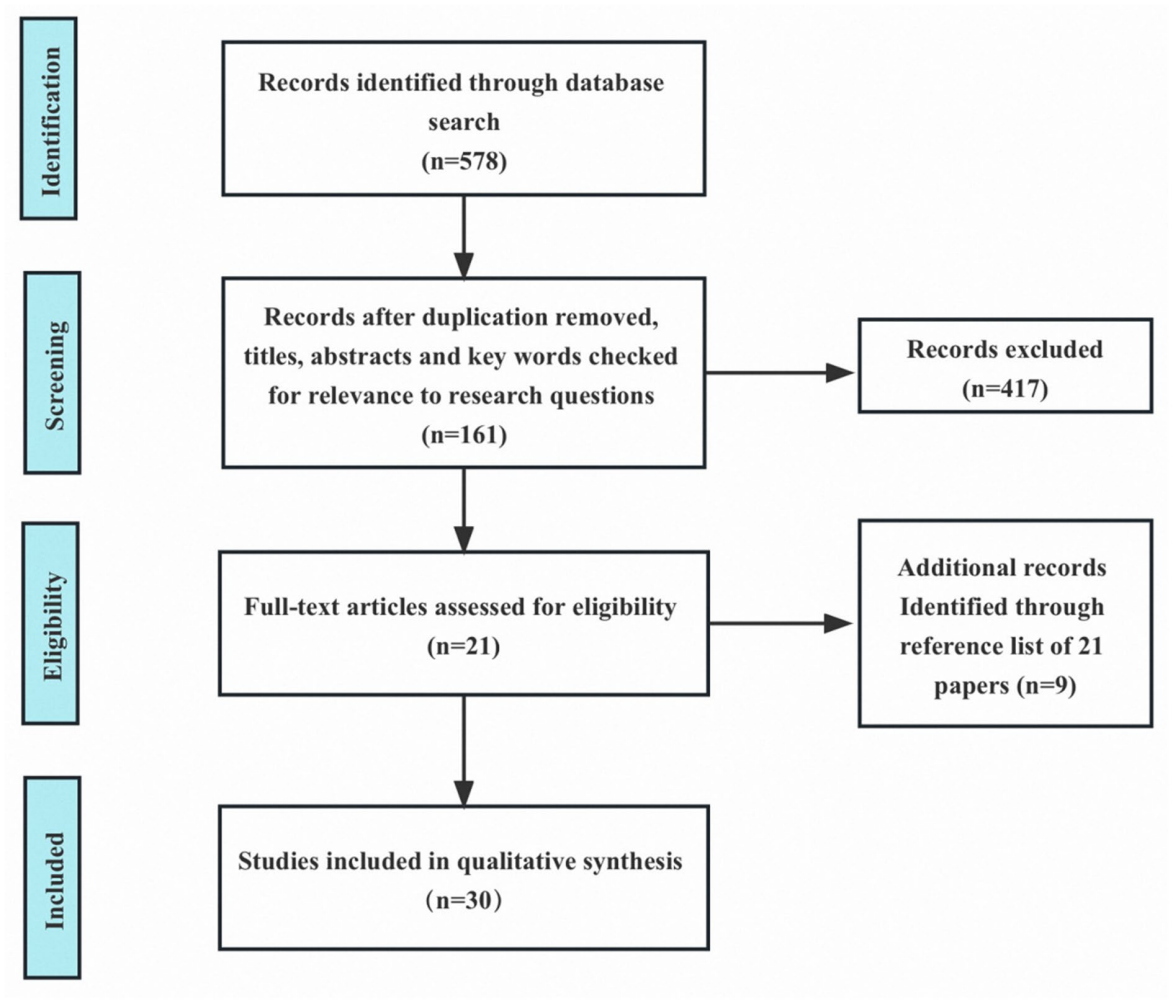
PRISMA flow diagram of the study selection process.

Before commencing the search, we developed inclusion and exclusion criteria prior to initiating our search. For a publication to be eligible for inclusion, it must meet the following four conditions: (1) focus on IP among doctoral students; (2) be written in English; (3) direct access to the full text; and (4) constitute a complete, peer-reviewed journal study. Any studies that fail to meet the above criteria were excluded from this review. At the same time, we did not impose any constraints on the year of publication to ensure a comprehensive and broad-ranging search approach, as a result, the oldest study was published in 1978 and the most recent study was published in 2023. The initial electronic database search yielded 578 articles that fulfilled the search criteria. After a thorough evaluation of titles and abstracts, publications, there were 161 articles left for further examination. To eliminate any potential duplication, we compiled a comprehensive catalog of these articles, including authors, titles, and publication years. After that, we then conducted a more detailed and in-depth analysis of the literature and assessed the full-text articles’ relevance based on their topic, research design, and findings. By employing this method, we were able to locate 21 articles that specifically addressed the subject of doctoral impostor phenomenon. Furthermore, we meticulously examined the reference lists within these articles and unearthed an additional 9 articles that related to impostor phenomenon among doctoral students. In the end, 30 articles met our inclusion criteria (as shown in Figure 1).

### 2.4. Data extraction

During the first stage, we collected essential information from each study, including the author, year of publication, country, sample size, discipline, study purpose, measure and results. To facilitate a systematic synthesis of the literature, we divided the above information into four main groups: (1) general overview (featuring author, publication date and country), (2) target group (including discipline and sample size), (3) study design (including study purpose, measure), and (4) results. In the second stage, we used descriptive and tabular formats to document our findings. Please refer to Table 1 for more detailed information.

**Table 1:** Summary of reviewed studies.

Elements		Number
Decade	1990’s	4
	2000’s	4
	2010’s	13
	2020’s	9
Country	America	19
	England	3
	Canada	2
	Russia	1
	Indian	1
	Brazil	1
	Israel	1
	Romania	1
	Finland	1
Discipline	Medicine	6
	Psychology	4
	STEM	3
	Clinical Psychology	2
	Others (eg:Arts, Business, Music, Management, English, Literacy)	7
Assessment tool	The Clance Impostor Phenomenon Scale (CIPS)	10
	The Hebrew version of the CIPS (HCIPS)	1
	The Doctoral Impostor Syndrome Scale (DIS)	1
	The Perceived Fraudulence Scale (PFS)	1
	The Zelen-O″Reilly Scale	1
	The Brief Symptom Inventory (BSI)	1
Study type	Survey	24
	Narrative review	2
	Qualitative	4

## 3. Results

Over the past 10 years or so, the issue of impostor phenomenon among doctoral students has become increasingly prominent in academic research, as reflected by the fact that two-thirds of the 30 papers reviewed were published between 2010 and 2023. Over half studies conducted in the United States and the remaining studies were conducted in the United Kingdom, Australia, Canada, Finland, Israel, Romania, and Russia. Additionally, a wide range of disciplines (i.e., management, clinical psychology, business, psychology, STEM, and medicine) studied this topic. Collectively, these studies provide valuable insights into the prevalence, causes, and consequences of IP among doctoral students across different contexts and fields of study.

### 3.1. Characteristics of the impostor phenomenon among doctoral students

First, empirical studies suggest that the pursuit of higher education does not necessarily foster a strong sense of self-efficacy among doctoral students. Self-efficacy refers to how doctoral students subjectively perceive and understand themselves as researchers and scholars, comprising their confidence and research capabilities. As the doctoral experience is widely recognized as both challenging and exhilarating, with the development of a high sense of self-efficacy playing a pivotal role in ensuring a successful doctoral pursuit. Despite entering programs with a variety of background experiences, motivations, ranges of academic and social situations, and capacities for research and scholarly productivity (Nettles and Millett, 2006), some doctoral students may not always feel confident in their research abilities or motivated to pursue higher academic aspirations. In particular, many doctoral students cannot view themselves as talented and competent students, after successfully completing baccalaureate and master’s degrees (Craddock et al., 2011). Throughout their doctoral studies, these students experience a pervasive sense of intellectual inadequacy, often believing that they lack the necessary skills for academic research. Students with IP focus on the recognition of others and external feedback instead of measuring their ability by what they have actually achieved. They may maintain a sense of relative value as long as the admiration of others exists. However, when positive feedback is not received, their good sense of self will then plummet (Langford and Clance, 1993).

Second, doctoral students with IP have a more negative attributional style. Specifically, the impostor phenomenon describes the self-attribution of success to luck and interpersonal skills rather than to intelligence and ability (Sightler and Wilson, 2001). Evidence suggests this presence of impostor characteristics among a group of 90 graduate students in clinical psychology programs. The survey revealed that those who experienced IP tended to attribute failure outcomes internally and globally. They also had an external locus of control in situations with successful outcomes and an internal locus of control for failure outcomes. This is because they cannot internalize success due to their inherent ability. When they are unsuccessful, they are likely to ascribe the reasons to factors such as effort (Niles, 1994). Simply put, participants tended to attribute their successes to external factors and blame themselves for their failures. According to attribution theory suggests that, attributions are a function of expectations. When a successful outcome is perceived as unexpected, this outcome is likely due to attribution to temporary external causes, such as luck or effort (Deaux, 1976). Thus, it appears that the way doctoral students attribute success and failure, as well as low expectations of success, is also, to some extent, a psychological projection of insecurity about self-competence.

Third, doctoral students with IP can fall into an abyss of fear. On the one hand, doctoral students face a variety of assessed such as publications, grants, qualifying examination, and their dissertations. Pursuing a Ph.D. degree is a long and challenging journey, requiring individuals to overcome several barriers. There are four general tasks of transition and initial socialization into graduate student life and the future career common to most doctoral students: gain intellectual competence, learn about the realities of life as a graduate student, learn about the profession for which one is preparing, integrate oneself into the department (Golde and Dore, 2001). Completing these challenges means being continuously evaluated by others and requiring a lot of time and effort. Doctoral students with IP often feel anxious and fearful of facing the dilemma of failing the challenge when their abilities and intelligence may be judged implicitly or explicitly. Indeed, these feelings are not caused by an external competitive environment or measures of success (e.g., publication or awards), but rather stem from a deep-seated personal denial of self that one is not effectively fulfilling the responsibilities that come with the role (e.g., as a doctoral student or academic researcher) (Nori and Vanttaja, 2022). On the other hand, others may have not yet discovered the deceptive behaviors of these doctoral students, causing them to live in constant fear of being exposed for the illusions they have created academically and intellectually. This fear is deeply unsettling and can push them into a painful cycle of impostorism (Gediman, 1985).

Fourth, doctoral students with IP may perform poorly academically and thus gradually drift off the academic track. When faced with a new task, individuals with IP usually doubt their ability to cope with the challenge and become overly concerned with their social image, which can lead to inefficient completion of the task (Maftei et al., 2021). For instance, regarding presentations and communication, doctoral students were consistently afraid of saying something foolish and being criticized by the audience. Similarly, they also worried that the articles they submitted for publication or those already published would be rejected by reviewers or readers (Chakraverty, 2020). Moreover, as they realize that their academic career may not be successful, they gradually lose their academic aspiration and adopt behaviors that are contrary to university expectations. A study by the Centre for Excellence in Teaching and Learning at the University of Waterloo (2016) found that graduate students with IP tend to have less contact with other students, struggle to effectively perform their duties as teaching assistants, and are less involved in practical activities, which can hinder their competence (Meurer and Costa, 2020). These characteristics can be used as indicators of impostor among doctoral students.

### 3.2. Factors contributing to IP among doctoral students

First, most doctoral students who have experienced IP indicate a lack of adequate academic preparation, specifically the ability of read, write, and academic think (Cisco, 2020). Overall, the doctoral stage of study requires individuals to be able to cope with unknown challenges and ultimately complete their studies (Kiley, 2009; Trafford and Leshem, 2009). One of the primary challenges that doctoral students face when entering graduate school is professional socialization. The process of transition from a learner to a researcher is akin to a rite of passage, requiring a liminal period of uncertainty, confusion, or doubt to achieve a change in identity (Van Gennep, 2019). In fact, many students may not understand the competencies requires for doctoral studies, how the educational process at the doctoral level works, or what it takes to successfully complete their studies (Golde and Dore, 2001).”Drifting” into doctoral education can exacerbate feelings of unease, incompetence, and low self-esteem (Nori and Vanttaja, 2022). In addition, doctoral students generally face multiple dilemmas such as difficult coursework, an increasingly competitive academic environment, and low levels of social support, which contribute to a challenging and stressful doctoral phase (McCauley and Hinojosa, 2020). When consciously struggling to meet the demands of their studies, they may consider themselves impostors.

Second, the impostorism of doctoral students is closely linked to their perceptions of role identity. Role identity serves as a frame of reference for assessing thoughts and actions and is the primary source for individuals to interpret and process life events and take further action plans (Vignoles et al., 2006). Max Weber regarded the academic profession at that time as a “calling” (Weber, 1994), highlighting the sacred status of academic work and its value implications. Currently, many Ph.D. students enter the academic world with enthusiasm but cannot break free from the continuous evaluation, monitoring of results, and glorification of excellence, some doctoral students might begin to demand too much from themselves and feel like an impostor who is not good enough for doctoral studies (Nori and Vanttaja, 2022).

Third, as far as the current educational environment is concerned, a culture of genius seems to permeate all stages of learning. The most direct effect of the cultural view of genius is to motivate individuals to try to hide. Individuals often alter their self-presentation to make themselves appear to have natural, primitive, and extraordinary intelligence. For the group of doctoral students, who have reached the top of the educational pyramid, external illusions about the intellectual talent of doctoral students induce them to fall into a strange circle. On the one hand, it is typical of them to feel they do not deserve to be in higher education and constantly need to prove themselves (O’Donnell and Tobbell, 2007). Thus, they will challenge themselves to difficult tasks and strive to achieve high standards of personal achievement. On the other hand, their confidence in their ability may be tested by unfamiliar academic practices making them feel vulnerable and marginalized (Housee and Richards, 2011). Out of low self-esteem, fear, and self-protection, they will try to avoid tasks that are beyond their ability to handle (Leary et al., 2000). This is in large part because they want to validate external illusions about their intellectual gifts are correct and use them to boost their self-esteem (Schubert and Bowker, 2017). However, it is undeniable that success at the doctoral level is not determined by a single factor, intelligence, but by a combination of multiple factors, which involves motivation and mental toughness at the individual level; school climate and faculty support at the organizational level; and institutional culture at the societal level. Ph.D. students with IP, by focusing too much on the role of intelligence in the learning phase, may attribute their failures to a lack of ability when they faced frustrations in their research, further exacerbating the sense of impostorism.

Fourth, IP is a habit-related phenomenon that can date back to early stages in the life course, i.e., deeply influenced by the family (Clance and Imes, 1978). IP feelings seem to be predicted by parenting patterns, suggesting family environments emphasizing grade-related achievement are associated with higher occurrences of IP (King and Cooley, 1995). Traditionally, in various studies on the influence of family background on children’s educational opportunities and outcomes, students from low-income families are generally considered to be in a disadvantaged position. However, studies have found that experiences of IP were evident, regardless of the home background (Nori and Vanttaja, 2022), though be differences in the degree of IP. For example, doctoral students from the working class or low-income families typically face more barriers such as financial pressures and cognitive limitations, in large part because they are the first generation of college students in their families. Ph.D. students from the middle- or upper-class families, on the other hand, are under tremendous pressure to succeed from their families, which leads them to feel insecure about their abilities and fear that they will not be able to surpass the success of the previous generation. In a word, both factors contributed to the experience of IP among doctoral students. Certainly, first-generation Ph.D. students tend to experience IP more frequently and to a greater extent (Terenzini et al., 1996). Thus, it is evident that the emergency of IP among doctoral students is influenced by deeper and more complex reasons. It cannot be solely attributed to individual imagination or perception but requires more comprehensive and scientific verification.

### 3.3. Correlation of IP with doctoral students’ mental illness

Previous studies have shown that IP is significantly associated with psychological problems among doctoral students. On the one hand, Ph.D. students with IP are accompanied by a variety of psychological problems including anxiety, fear, and depression (Craddock et al., 2011; Lane, 2015). These psychological problems can, in turn, lead to behavioral tendencies such as academic burnout, procrastination, or perfectionism. Poor academic performance and academic psychological problems can further contribute to the development of IP in doctoral students, generating a cycle. Specifically, discrepancies between reflected evaluations and one’s ideal self-image (what one would like to be) can lead to depression. Likewise, discrepancies between reflected evaluations and ones perceived standards can spawn anxiety and distressing emotions (Higgins, 1989). Due to impostors’ relentless pursuit of success and their difficulty accepting affirmation when they achieve and falling short when they fail, they are often under increasing stress and highly vulnerable to psychological problems. These psychological crises are often implicit and can subconsciously weaken an individual’s ability to function at the highest level, resulting in a gradual decline in academic performance and satisfaction (Hirschficld, 1982; Zorn, 2005; Whitman and Shanine, 2012; Pervez et al., 2020). On the other hand, IP can also act as a mediator, moderating the relationship between perfectionism or procrastination and anxiety. For example, a survey of 169 Russian college students indicated that imposter Phenomenon fully mediated the link between perfectionism and anxiety, whereas it served as a partial mediator between perfectionism and depression (Wang et al., 2019). Additionally, IP can effectively mediate the relationship between procrastination and anxiety (Maftei et al., 2021). Although some studies have generally highlighted the negative effects of IP on individuals’ psychological and behavioral performance and ignored its beneficial side, the above findings reveal that we are supposed to view IP rationally and take advantage of its positive effects.

### 3.4. Scales measuring IP

Overall, the Harvey Impostor Phenomenon Scale (HIPS) and the Clance Impostor Phenomenon Scale (CIPS) are two widely used measures for imposter feelings. Specifically, HIPS was constructed by Harvey in 1981 that aims to evaluate the individuals’ perception and cognition regarding IP. It consists of 14 items and scored on a scale ranging from 1 (not at all true) to 7 (completely true). Such measure was valided among 74 students and the internal consistency coefficient was 0.85 (Harvey, 1981). Subsequently, Clance developed the Clance Impostor Phenomenon Scale, and identified a three-factor model including self-doubts about ones’ own intelligence and abilities (Fake), tendency to attribute success to chance/luck (Luck), and the inability to admit a good performance (Discount) (Clance, 1985). It consists of 20 items and scored on a scale ranging from 1 (not at all) to 5 (fully). After examining the clinical and non-clinical samples, the scale was found to have a Cronbach coefficient between 0.84 and 0.96 (Clance, 1985). In summary, both these two scales have a stable factor structure and are practical tools for measuring IP among clinical or no-clinical sample, and confirmed by previous studies (Edwards et al., 1987; Chrisman et al., 1995). In comparison, CIPS is capable of independently identifying impostors and non-impostors in both clinical and non-clinical samples. Therefore, CIPS is more sensitive and reliable, and is considered as the “gold standard” for measuring IP (Holmes et al., 1993).

In terms of specific applications of the scale, the CIPS has been used to assess the impostor phenomenon in various population, including female Hebrew students (Yaffet, 2020), Romanian psychology students (Maftei et al., 2021), and others. For the measurement of IP among doctoral students, Nori and Vanttaja developed the Doctoral Impostor Syndrome (DIS) scale, according to a survey in 2015 that included 1,694 Finnish doctoral students. As the original questionnaire covered a broad range of topics and consisted of 70 items, Nori et al. simplied the questionnaire and selected 10 items in relation with IP of doctoral students in the DIS scale, such as their family background, social network, professional socialization, and personal abilities (Nori and Vanttaja, 2022). In summary, the DIS scale provides a more accurately and scientifically grounded assessment of IP among Finnish doctoral students. Given the lack of measurement scales tailored to doctors students’ IP, researchers can draw on the methodology employed by Nori to develop other scales that explore doctors students IP, thereby further enriching the fields’ findings.

## 4. Discussion

Since the concept of impostor phenomenon was introduced, attention to it has increased dramatically in the past decade, both in academic research articles and in the popular media (Feenstra et al., 2020). In early studies, psychologists and social scientists primarily focused on individual traits and behaviors, exploring the impact of IP on an individual’s mental health and career development. For example, much of the research on IP focused on the early stages of an individual’s career, such as medical interns, and has explored the dilemmas they encounter in their professional development. Studies on similar groups suggests that IP is not unique to a particular culture but may be a product of professional characteristics, namely, IP plays an important role in successful career development. As time went on, researchers began to shift their focus toward the social, cultural, and institutional dimensions of IP and actively explored methods to address it. These studies have not only revealed the prevalence of IP but also provided important insights into its negative impact on individuals and groups. There is no doubt that the research conducted over the past few decades has deepened our understanding of IP and equipped us with frameworks and strategies to recognize, comprehend, and address this psychological phenomenon.

In recent years, IP among doctoral students has gradually become a research hotspot in academia. This is due to the profound changes in the global higher education environment, the pervasive crisis of uncertainty worldwide, and the continuous conflicts and challenges individuals face. For doctoral students, the most prominent issue is academic career development. In the past, academic profession was regarded as a “Linear Pipeline” for post-degree career development, while entering a non-academic profession was seen as a “Leakage of the Pipeline” (Fuhrmann et al., 2011). Nowadays, tenure opportunities are becoming less and less available, the academic career market is saturated, and new modes of selection and recruitment have been introduced (Benz et al., 2020). There is data indicating that in 11 countries, about 10% of doctoral graduates found jobs that were not related to their specialization or only required a lower degree (Auriol, 2007). Graduates have realized that a Ph.D. degree is no longer a passport to a lifelong job. They need to acquire transferable and flexible skills that can in turn adequately condition them to be prepared for the changing academic market (Delanty, 2001). Doctoral students thus oscillate between the development of a professional identity (which is critical to career success) and that of a professional scholar (which is critical to academic success), moving between different roles and expectations and trying to form both identities (Austin and McDaniels, 2006). In summary, with the changing academic career landscape and increasing competition, the psychological pressures on doctoral students have intensified, which exacerbate doctoral student’s perception that they do not have the ability to reach the requirements of academic career, and finally leading to a growing number of cases of IP among doctoral students.

The research reviewed and synthesized nearly 40 years of research, theories, and frameworks on the impostor phenomenon. After conducting a systematic and comprehensive review, we have found that previous research on IP among doctoral students has explored various key areas. We examined common experiences, emotions of self-doubt, and cognitive patterns among doctoral students, explored various dimensions of mental health, including stress, anxiety, depression, and other psychological indicators relevant to the doctoral student population. In addition, we summarized existing literature and research findings to determine whether individuals who experience the impostor phenomenon are more prone to mental health issues or vice versa. Last, we focused on identifying and examining existing scales used to assess the impostor phenomenon and evaluated various scales specifically designed to measure IP among doctoral students. The research findings provide updated insights into the impostor phenomenon. And one of the contributions of our study is to summarize the characteristics of doctoral students experiencing IP, including low self-efficacy, a tendency toward negative attributions, heightened anxiety, and lower academic performance. In conclusion, within the existing research, IP is indeed described as a combination of various concepts, representing a comprehensive description. We tend to view it as a distinct psychological pattern in which individuals doubt their achievements, believe their abilities do not match their accomplishments, and live in fear of eventually being exposed. The studies also highlight the impact of the impostor phenomenon on the mental health of doctoral students and explore the potential relationship between mental health status and IP. Additionally, the research results provide an overview of scales that can be used to specifically measure IP among doctoral students, offering convenience for future research. Overall, the research findings contribute to a deeper understanding of the impostor phenomenon and its effects on the well-being of doctoral students.

The former studies on IP among doctoral students have indeed made significant contributions. However, there are certain gaps that require addressing. For instance, research on interventions still needs to be enriched. Existing studies point out how to curb IP among doctoral students, for example, by considering that the more confident doctoral students are in their abilities, the more likely they are to succeed in their academic research. Therefore, supervisors can help doctoral students correctly perceive their abilities when they express imposter emotion (Coryell et al., 2013). If individuals with IP attribute positive outcomes to luck, their misattributions and perceptions of achievement can be changed by identifying stable, positive personality traits associated with their success (Wang et al., 2019). It should be noted that the emergency of IP among doctoral students is not only about individual academic literacy but also directly linked to the mismatch between the purpose of doctoral education, the student’s vision, and their actual situation both in and out of academia. Accordingly, when suggesting countermeasures, we should not only stop at a single surface level, but also place it in a more macro context for comprehensive consideration and further suggest targeted recommendations. For example, how to prepare Ph.D. students for multiple career paths, and how to enhance the adaptability of Ph.D. students from academic to non-academic tracks are all questions that deserve careful consideration in future research (see Table 2).

**Table 2:** Key characteristics of reviewed studies.

Author (Year)	Country	Sample size	Discipline	Study purpose	Measure	Main findings
Niles (1994)	America	*N* = 90	Clinical Psychology	If graduate students in clinical psychology programs differ from Ph.D. graduates and experienced clinicians.	Professional Attributional Style Questionnaire; the Zelen-O″Reilly Scale.	Impostors scored significantly higher on measures of external locus of control than non-impostors
Chrisman et al. (1995)	America	*N* = 269	Psychology	Compare the PFS with the CIPS.	PFS; CIPS.	Both CIPS and PFS had similar internal consistency reliability. CIPS is a more useful instrument for clinical and research purposes.
Ewing et al. (1996)	America	*N* = 269	NA	Examine whether IP exists among African American graduate and professional students.	Online questionnaire.	There was no correlation between racial identity attitudes and IP.
Henning et al. (1998)	America	*N* = 477	Medicine	Examine the severity of perfectionism and the IP in health profession students, and assess the relationship between these personality traits and students’ psychological adjustment.	The Brief Symptom Inventory (BSI); The Multidimensional Perfectionism Scale; CIPS.	Strong associations were found between psychological distress, perfectionism and impostor feelings and these character traits were stronger predictors of psychological adjustment than most of the demographic variables associated previously with distress in health professional students.
Golde and Dore (2001)	America	*N* = 4,114	Arts and science	Provide a snapshot of the experiences of doctoral students in the arts and sciences.	The Survey on Doctoral Education and Career Preparation.	Many students did not understand what doctoral study entails, how the process works, or how to navigate it effectively.
White (2001)	America	*N* = 158	Clinical Psychology	Explore the nature of perceived fraudulence from a perspective informed by self-psychological theory.	Online questionnaire.	One dimension of self-cohesion (goal instability), as well as shame, are significant predictors of perceived fraudulence in this sample.
Castro et al. (2004)	America	*N* = 213	Psychology	Examine individuals who were parentified as children are more likely to report impostor feelings in adulthood.	The Parentification Questionnaire; CIPS.	The impostor phenomenon can be explained, in part, as a significant long-term effect of childhood parentification.
Gibson-Beverly and Schwartz (2008)	America	*N* = 170	NA	Examine the utility of attachment and entitlement as predictors of the impostor phenomenon in female graduate students.	CIPS; Entitlement Attitudes Scale (EAS); Experiences in Close Relationships Scales–Revised (ECR-R).	Individuals with high levels of self-reliance/self-assurance entitlement are able to associate positive feedback with stable internal attributes.
Cope-Watson and Betts (2010)	Canada	*N* = 2	NA	Connect the theoretical frameworks around IP to our experiences as women graduate students in a doctoral program.	Autoethnography.	The tensions created between an externally imposed perception of academic ability and an internally imposed perception of self-deficit can contribute to feelings of inadequacy.
Craddock et al. (2011)	America	*N* = 6	NA	Explore IP feelings.	Interview.	IP feelings were a normal part of graduate study.
Knights and Clarke (2014)	England	NA	NA	Provide a brief examination of the literature on identity, insecurity and academic selves, particularly those in business schools.	Semi-structured interview.	Academic settings in higher education can trigger impostor phenomenon.
Lane (2015)	America	*N* = 29	Business, Music, Engineering, Psychology, Counseling	Qualitatively explored IP.	Survey; interview.	Most offered specific examples of experiences consistent with the IP.
Aubeeluck et al. (2016)	England	NA	Medicine	Debate an issue.	Article review.	70% of the participants had frequent to intense experiences of IP.
Neureiter et al. (2016)	America	*N* = 212	Psychology	How IP functions in career development.	Questionnaire.	IP was relevant to career development in different ways at different career stages.
Schubert and Bowker (2017)	Canada	*N* = 304	Psychology	Examined how the Impostor Phenomenon relates to multiple dimensions of self-esteem.	CIPS, the Rosenberg Self-Esteem Scale.	People with low self-esteem were especially vulnerable to impostor feelings.
Villwock et al. (2016)	America	*N* = 2,612	Medicine	Describe levels of burnout and IP in medical students, and recognize demographic differences in those experiencing burnout and IS.	Survey.	Almost a quarter of male medical students and nearly half of female students experience IS and IS was found to be significantly associated with burnout indices.
Bothello et al. (2019)	England	NA	Management	One where the induction rituals both formal and informal are in many ways misaligned with the multi-dimensional roles of our profession.	NA	These students are noticeably more self- assured and motivated than their peers and less likely, we find, to suffer from feelings of imposterhood.
Chakraverty (2020)	America	*N* = 120	STEM	Explore different themes related to impostor phenomenon, as experienced by graduate students and postdocs in science, technology, engineering and mathematics (STEM) fields.	Open-ended survey.	Those experienced the impostor phenomenon in graduate school have attributed it to one’s good luck and ability to pretend as well as other’s kindness and poor judgment of skills.
Cohen and McConnell (2019)	America	*N* = 1,476	Arts, Sciences, Business, Library science	Examine the relationship between perceived characteristics of graduate school program environments and students’ impostor feelings.	The 2013 Graduate Student Stress and Coping (GSSC) survey.	Students’ perceptions of lower-quality mentorship, increased competition, and increased isolation are associated with more frequent impostor fears.
Gottlieb et al. (2019)	America	*N* = 18	Medicine	Analyze the existing literature on IS among practicing physicians and physicians in training.	Article review.	Gender, low self-esteem, and culture were associated with higher rates of IS.
Wang et al. (2019)	Russia	*N* = 169	Management, Economics, Business Informatics, Law, Program Engineering	Understand factors that put individuals with this particular profile at risk is important.	Mediated and moderated the link between perfectionism and psychological distress were examined.	IP fully mediated the link between perfectionism and anxiety, whereas it served as a partial mediator.
Chakraverty (2020)	Indian	*N* = 90	STEM	Explored various facets of impostor phenomenon in STEM.	Interview.	Progress and public recognition, comparing oneself with others, developing skills: public speaking and scientific writing, application of new knowledge, and asking for help triggered IP.
Cisco (2020)	America	*N* = 42	Art, English, Literacy, Math, Music, Science	In what ways are postgraduate students experience impostor phenomenon?	CIPS; interview.	The majority of the participants experienced IP and felt academically unprepared.
Lee et al. (2020)	America	*N* = 959	STEM, Medicine	How IP relates to self-evaluation.	CIPS.	Different types of IP included more strategic self-presentations of ability, and the defining feature of IP might be fear rather than self-doubt.
Meurer and Costa (2020)	Brazil	*N* = 181	Business	Analyze the relationship between IP and the academic behavior of postgraduate students in business.	Meurer and Costa Scale of Academic Behaviors Stricto *Sensu* (MCSABSS).	56.15% experienced high and intense impostor syndrome and presented high levels of distress.
Pervez et al. (2020)	America	*N* = 113	Management	Examine the prevalence of depression and anxiety Symptoms in management doctoral students.	Online questionnaire.	Management doctoral students experienced depression and anxiety symptoms at significantly higher rates.
Yaffet (2020)	Israel	*N* = 248	NA	Investigate the psychometric properties of the Hebrew form of the CIPS (HCIPS).	HCIPS, CIPS.	HCIPS was a sound instrument for assessing impostor feelings among female Hebrew-speaking students.
Maftei et al. (2021)	Romania	*N* = 130	Psychology	Explore the prevalence of the impostor syndrome and its associated factors.	CIPS.	56.15% experienced high and intense impostor syndrome and presented high levels of distress.
Nori and Vanttaja (2022)	Finland	*N* = 1,694	NA	Focus on the prevalence of IP among Finnish Ph.D. students.	DIS.	Impostor feelings occurred most commonly. The DIS formed an internally congruent gage.
Neufeld et al. (2023)	Canada	*N* = 1,450	Medicine	Explore the prevalence of IP among the students.	CIPS.	Students who are more self-determined (both in general and in medical school), and whose basic psychological needs are more supported in their medical program, will experience less frequent and severe IP symptoms.

Moreover, the existing research on IP among doctoral students offers many exciting possibilities for further investigation. Chance’s study highlighted that IP, especially the internal experience of intellectual phoniness, is prevalent and intense among high achieving women (Clance and Imes, 1978). Indeed, previous studies have suggested two types of IP: “True” impostors characterized by the negative self-views associated with the construct definition, and more “strategic” impostors who seem to be less encumbered by self-doubt. It is assumed that “strategic impostors” are characterized by a form of deliberate self-presentation (Leonhardt et al., 2017). Therefore, it is necessary to treat IP among doctoral students with more caution. In the current educational environment, Ph.D. students undergo a heavy competitive selection process to gain admission, which serves as evidence of their ability to complete their studies. Thus, they are in a very different situation from genuine impostors who lack ability and intellectual talent. The case in point is researchers can explore the hypothesis that IP is driven not only by suspicion but also by fear (Lee et al., 2020). By creating novel scales and distinguishing various types of impostors, researchers can examine how fear and suspicion contribute to the development of IP among doctoral students. Additionally, it is crucial to consider the impact of cultural and social backgrounds on doctoral education systems and how they shape doctoral students’ experiences with the Impostor Phenomenon. Conducting comparative or country-specific research can help identify differences in these experiences and challenges across districts and geographical locations, contributing to the advancement of global higher education. By continuing to explore new avenues of research, we can better understand the impostor phenomenon among doctoral students.

## 5. Limitations

There are some limitations to this study. First, it is important to note that this study exclusively focused on English-language publications, potentially overlooking the extensive utilization of other languages such as Spanish, French, and Russian in academic publishing and dissemination. Consequently, there is a possibility of incomplete incorporation of research findings. Second, the scoping review aimed to provide a broad overview of the existing evidence base related to a particular topic, without considering the quality of the literature. However, not assessing the quality of the literature may lead to a lack of accuracy in the conclusions drawn from them. Furthermore, the conclusions drawn from these studies may not be verified due to the lack of actual inquiry. Therefore, it is important for future reviews to consider the quality of the literature when conducting a scoping review, to ensure that all relevant studies are included and that the conclusions drawn from these studies are accurate and reliable.

## 6. Conclusion

This review explored four key aspects of IP in doctoral students, including manifestation characteristics, reasons for its emergence, correlation with psychological problems, and scales for evaluation. In conclusion, current research indicates that this population may be particularly susceptible to experiencing feelings of intellectual fraudulence and self-doubt. Doctoral students with IP often struggle with low self-efficacy and suffer from negative attributions and fears, which can hinder their academic performance and derail their academic paths. Moreover, factors such as inadequate academic preparation and support, and the influence of the genius culture can all contribute to the emergence of IP among doctoral students and may have negative impacts on academic progress and mental health. Research has shown that symptoms of anxiety, depression, and burnout are common among this population, and may be related to feelings of imposture as well as other factors. Evidence also suggests that poor mental health can have negative impacts on academic success, and that interventions aimed at improving mental health may also lead to improvements in academic outcomes. Although there are two commonly used scales, HIPS and CIPS, that can measure the impostor phenomenon (IP), the DIS scale developed by Nori is considered more appropriate in assessing IP among doctoral candidates specifically. It is important to improve doctoral students’ comprehension of IP to address this issue and promote their success in academia. By conducting further research and collaborating with stakeholders, we can work together to create a more equitable and inclusive learning environment that empowers doctoral students to achieve their academic and professional goals. Overall, it is critical that we continue to prioritize the well-being and development of doctoral students in our efforts to improve the impostor phenomenon.

## Data availability statement

The original contributions presented in the study are included in the article/supplementary material, further inquiries can be directed to the corresponding author.

## Author contributions

YW made significant contributions to the conception and design of this work, as well as interpreting the data, revising important intellectual content, and writing the manuscript. Meanwhile, WL oversaw the study’s design, participated in data collection and analysis, and contributed to revision of the manuscript. All authors were involved in the manuscript’s final version, which they approved.
